# Dietary intake of branched-chain amino acids and pancreatic cancer risk in a case-control study from Italy – ERRATUM

**DOI:** 10.1017/S0007114523001010

**Published:** 2023-12-14

**Authors:** Marta Rossi, Federica Turati, Panagiota Strikoudi, Monica Ferraroni, Maria Parpinel, Diego Serraino, Eva Negri, Carlo La Vecchia

**Details of correction:** reverse order of first and second authors.

**Existing text:** Federica Turati, Marta Rossi

**Corrected text should read:** Marta Rossi, Federica Turati

